# Physical Exercise and Dietary Supplementation in Middle-Aged and Older Women: A Systematic Review

**DOI:** 10.3390/jcm12237271

**Published:** 2023-11-23

**Authors:** Juan Carlos Sánchez-García, Daniel López Hernández, Beatriz Piqueras-Sola, Jonathan Cortés-Martín, Andrés Reinoso-Cobo, María José Menor-Rodríguez, Raquel Rodríguez-Blanque

**Affiliations:** 1 Research Group CTS-1068, Andalusia Research Plan, Junta de Andalucía, 18014 Granada, Spain; jsangar@ugr.es (J.C.S.-G.); bpiquerassola@gmail.com (B.P.-S.); rarobladoc@ugr.es (R.R.-B.); 2 Department of Nursing, Faculty of Health Sciences, University of Granada, 18071 Granada, Spain; 3 Department of Nursing, Military School of Health, 28047 Madrid, Spain; dlophe3@mde.es; 4 Virgen de las Nieves University Hospital, 18014 Granada, Spain; 5 Department of Nursing and Podiatry, Faculty of Health Sciences, University of Malaga, 29071 Malaga, Spain; andreicob@uma.es; 6 Área Sanitaria Santiago de Compostela-Barbanza, Subdirección de Humanización y Atención a la Ciudadanía, 15706 Santiago de Compostela, Spain; mariajosemenor@hotmail.com; 7 San Cecilio University Hospital, 18071 Granada, Spain

**Keywords:** menopause, osteoporosis, exercise, dietary supplements, aging

## Abstract

With the aging of the population in developed countries, the number of middle-aged and older women is progressively increasing. During this stage, women suffer from a number of signs and symptoms that could be reduced or treated with physical exercise and dietary supplements. The main objective of this study was to analyse the benefits of exercise and dietary supplements during menopause. Materials and methods: A systematic review of the scientific literature was performed according to the PRISMA 2020 protocol, searching the PubMed, Cochrane, Scopus, and WOS databases. Studies that met the inclusion criteria were assessed for methodological quality using the PEDro or AMSTAR-2 scales. Results: The searches yielded a total of 104 results, of which 10 were selected, with methodological quality ranging from fair to excellent. Each article examined the combination of a dietary supplement plan versus a placebo; plus an exercise routine versus another routine or a sedentary lifestyle. The results showed the benefits of combining a nutritional supplementation plan with an exercise routine during menopause. Conclusions: The practice of weekly strength and endurance exercises, together with the consumption of certain dietary supplements, may be a good resource for coping with menopause in a healthy way.

## 1. Introduction

Menopause is a process that marks the end of the reproductive phase in women. This process usually occurs between the ages of 45 and 55 [1]. Menopause means both that the ovaries stop producing eggs and that there is a sharp decline in the production of female hormones such as progesterone and oestrogen [2,3]. The climacteric is the transitional period between the reproductive stage and menopause [4], during which changes occur in a woman’s body: increased blood pressure (BP) due to decreased arterial elasticity and increased stiffness and, thus, increased risk of stroke; decreased glucose tolerance and, thus, increased risk of type II diabetes; coagulation problems; increased levels of low-density lipoprotein (LDL) and decreased levels of high-density lipoprotein (HDL); weight gain; and metabolic slowing [2,5,6].

Menopause is directly linked to osteoporosis, with oestrogen deficiency being a major contributor to the increased risk of this disease [6], which results in a decrease in bone mineral density (BMD) due to osteoclast-mediated bone resorption and an increased risk of fractures. The SWAN study links early menopause (45 years or younger) with cardiovascular disease, stroke, and death from coronary heart disease. An elevated BMI at menopause is associated with metabolic syndrome and cardiovascular risk factors [7,8].

Treatment for middle-aged women varies depending on how the woman is affected and can include a wide repertoire of medications. The most common is hormone replacement therapy (HRT), either with oestrogen alone (after a hysterectomy) or with oestrogen and progestin (in the presence of the uterus to prevent endometrial hyperplasia). HRT is a treatment that contains female hormones, and it is most commonly used to treat common symptoms of menopause, including hot flashes and vaginal discomfort. Hormone therapy has been shown to prevent bone loss and reduce the risk of fractures in postmenopausal women. It focuses primarily on replacing the oestrogen that women stop producing after menopause. There are two main types of hormone therapy: systemic hormone therapy (in the form of a pill, skin patch, ring, gel, cream, or spray, which usually contains a higher dose of oestrogen that is absorbed and distributed throughout the body), that can be used to treat all of the common symptoms of menopause. And low-dose vaginal products (in the form of creams, tablets, or rings, minimizing the amount of oestrogen absorbed by the body). For this reason, low-dose vaginal preparations are generally used only to treat vaginal and urinary symptoms of menopause [9]. Other treatments include antidepressants for depression; gabapentin may be indicated for hot flushes; depending on cholesterol levels or increased blood pressure, statins and antihypertensives are prescribed; and, of course, drugs to treat osteoporosis, including bisphosphonates, raloxifene, and denosumab [10,11,12].

Exercise is a promising intervention not only for the benefits it offers during menopause, but also for the benefits it can offer during aging or at any stage of life [13,14]. High-intensity interval training (HIIT) programs have been shown to significantly reduce body weight and total and abdominal fat mass in women. These effects have been observed to be more pronounced in premenopausal women than in postmenopausal women. In addition, cycling in HIIT has been found to be more effective than running, especially in postmenopausal women. For optimal results, it is recommended that training lasts more than 8 weeks, with at least three sessions per week [14].

Endurance exercise is defined as 20–50 min of exercise at a heart rate (HR) of 120–160 beats/min. Exercises include walking, running, swimming, dancing, cycling, jumping, and tai chi [15,16].

Resistance exercise programs include all exercises that focus on building strength and/or hypertrophy of muscle. These exercises are in the range of 10–15 repetitions at higher or lower intensities, depending on several variables such as the weight used, intensity, training volume, and the equipment used, which may be weights such as dumbbells, pulleys, or other machines, or even body weight itself, as in the case of calisthenics [15].

HIIT is a type of training in which the subject performs some high-intensity exercise (e.g., sprinting, running at an intense pace, stationary cycling, CrossFit) for a time interval that is shorter, equal, or longer than the rest period, depending on the physical condition of the subject [14,15].

On the other hand, there are dietary supplements, which are nutrients or parts of nutrients taken in addition to the diet to improve health or athletic performance [17]. Some may be helpful during menopause and some are widely known to be used during this stage [18], while others are more focused on health benefits or improving athletic performance, such as creatine, protein powder, omega-3, etc. [19].

With the growing interest in sport and the consumption of dietary supplements [20], in addition to the ageing of the population in developed countries, it would be interesting to study which type of exercise is most appropriate during menopause, which dietary supplements are really an effective aid among the vast supply on the market, and, above all, to show the benefits of both exercise and supplementation during this stage of life.

Our PICO question would be: “In women experiencing menopause (Population), does the implementation of exercise and dietary supplements (Intervention) compared to a control group or no specific intervention (Comparison) have a significant impact on reducing menopausal symptoms and their negative effects (Outcomes)?”.

The primary objective of the present study is to discuss the benefits of exercise and dietary supplements during menopause.

The secondary objectives are to determine whether the relationship between dietary supplements and exercise reduces the negative effects of menopause and to investigate which dietary supplements and exercise may be beneficial during menopause. The main objective of the present study is to discuss the benefits of exercise and dietary supplements during menopause.

## 2. Materials and Methods

### 2.1. Design of the Study

This study is a systematic review of the published scientific literature on the benefits of exercise and dietary supplementation in menopausal women. It was conducted in accordance with the requirements of the Preferred Reporting Items for Systematic Reviews and Meta-Analyses (PRISMA 2020) review protocol. It is registered on the PROSPERO website (http://www.crd.york.ac.uk/PROSPERO/, accessed on 15 May 2023) and its registration number is (CRD42023423824).

### 2.2. Eligibility Criteria

Inclusion Criteria

- Randomised and non-randomised clinical trials.
- Meta-analysis.
- Studies carried out in adult menopausal women, according to the guidelines of the W.H.O., who are not on hormone replacement therapy; healthy or with menopausal complications: osteoporosis, dyslipidaemia, hypertension, obesity, etc.
- Articles published less than 5 years ago, regardless of language.

Exclusion Criteria

1. Reviews, both systematic reviews and review articles. We did not exclude articles that were systematic reviews with meta-analysis. Studies conducted in patients with other serious diseases that could affect the results (e.g., cancer).
2. Studies in which menopause and exercise or dietary supplements did not appear as keywords in the abstract or title; or in which both interventions did not occur in the study population.
3. Studies of low methodological quality: PEDro scale score less than 4 points or low AMSTAR-2 scale score.

### 2.3. Search Strategy and Information Sources

A literature search was performed independently by two researchers (J.C.S.-G. and R.R.-B.) between November 2022 and February 2023 in the following databases: PubMed, Web Of Science, Scopus, and Cochrane.

The terms used in the search are listed in Table S1. The search strategy used (Table S2) was the same in all four databases and used the following keyword format: (menopause) OR (natural language) AND (“dietary supplements”) OR (natural language) AND (exercise) OR (natural language).

To limit the search, we applied both publication date filters, limiting the search to no more than 5 years, and study type filters, where available, limiting the search to randomized and non-randomized trials, meta-analyses, and articles.

An additional eight searches were performed, two in each database, using the following strategy:

- (menopause) OR (natural language) AND (“dietary supplements”) OR (natural language).
- (menopause) OR (natural language) AND (exercise) OR (natural language).

No results were found, or the very few results found were not of interest for the study, so it was decided not to include them in the search strategy.

Table S2 shows the different search strategies in each database, date, results obtained, and articles selected in each one.

### 2.4. Selection Process of the Studies

Two investigators (D.L.H. and J.C.-M.) independently selected the studies to be included in the results by reading the titles and abstracts. Studies were selected if, in addition to meeting the inclusion criteria, they provided recent and conclusive evidence that exercise and/or dietary supplementation can benefit menopause or counteract the negative effects of menopause in women.

Studies that did not meet the inclusion criteria or showed inconclusive evidence were excluded from the results.

### 2.5. Data Extraction Process

Data extraction was performed independently by two investigators (D.L.H. and J.C.-M.) after reading the full text of the studies. The investigators independently read the full-text manuscripts and extracted the following information from each study: authors, country, year, aim of the study, type of study, sample, characteristics of the participants (age, previous pathologies, etc.), duration of the study, main findings and conclusions. In the case of randomized trials, information on the intervention, comparison, results, and conclusions were extracted.

### 2.6. Methodological Quality Control Tools

The methodological quality was assessed independently by two investigators (J.C.S.-G. and R.R.-B.) using two tools or questionnaires:

For meta-analyses, the AMSTAR-2 (A Measurement Tool to Assess Systematic Reviews) questionnaire was used [21], which has 16 items and classifies studies into 4 confidence levels according to the score: High—provides an accurate and complete summary of the results of available studies; Moderate—has weaknesses but may provide an accurate summary of the available studies; and Low—may not provide an accurate and complete summary of the available studies. This rating was chosen as a cut-off for including or excluding studies. Finally, Critically Low—it is not reliable.

For clinical trials, we used the PEDro (Physiotherapy Evidence Database) questionnaire [22], which has eleven items (ten of which are scored) and classifies studies into four levels of methodological quality based on the score obtained: excellent (9–10 points), good (6–8 points), fair (4–5 points)—this score was chosen as a cut-off for including or excluding studies—and finally, poor (less than 4 points).

## 3. Results

Ten articles were included in the systematic review and were selected based on a number of key characteristics:

- Scientific evidence no older than 5 years. Meta-analysis or clinical trials.
- The study sample is healthy middle-aged women or women with middle-aged pathologies (due to or common during menopause) such as osteoporosis, dyslipidaemia, hypertension, and sarcopenia, among others.
- They provide evidence for the benefits of a specific exercise program, a dietary supplement, or a combination of both.

After reading the full text of the articles selected for the systematic review, the following information was extracted from them:

- Author, country, and year.
- Type and duration of the study.
- Sample and main characteristics of the sample.
- Aims of the study.
- Main findings.
- Conclusions.

**Figure 1:**
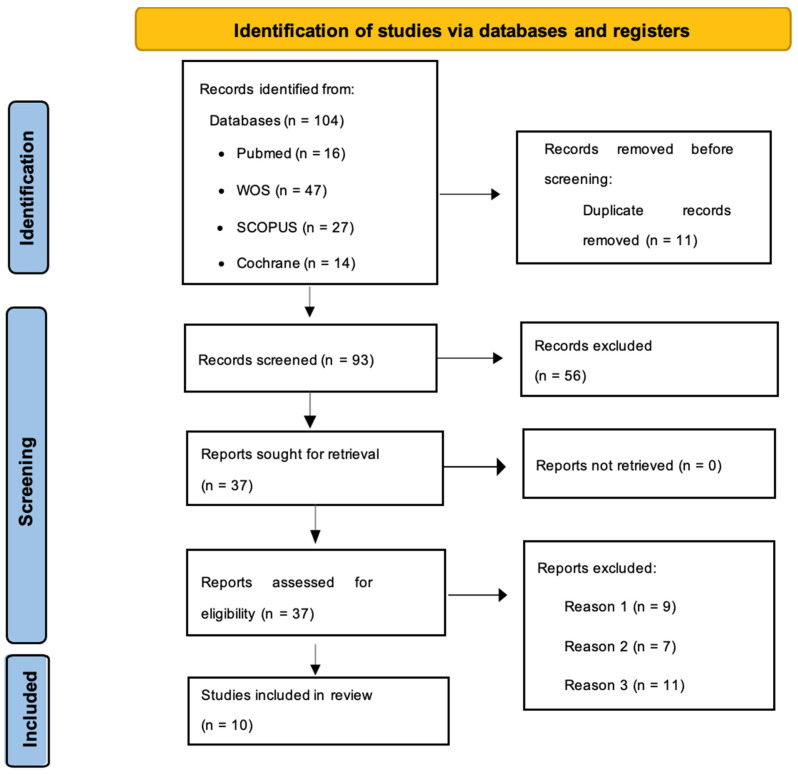
Flow diagram. Reason 1: Studies conducted in a population exercising or taking supplements separately, not both interventions together. Reason 2: The study did not answer the investigator’s question. Reason 3: Did not meet inclusion criteria or met exclusion criteria.

Details regarding the inclusion and exclusion of studies at each stage are provided in the flow chart (Figure 1).

The results of these studies are shown below in tabular form (Table 1).

The scores obtained for each of the articles used in the systematic review when calculating the risk of bias are shown in Table 2 and Table 3.

For the clinical trials, use was made of the PEDro scale [22], where the exclusion cut-off was decided at a score of less than 6 points, which corresponds to evidence rated as fair or poor (Table 2).

For meta-analyses, the AMSTAR-2 scale [21] was used, where the exclusion cut-off applied to articles that were rated as “low evidence” or “critically low” (Table 3).

## 4. Discussion

Physical exercise has beneficial effects during all stages of a woman’s life. During menopause, this manuscript has compiled data indicating that it can also combat many of the associated symptoms, providing benefits for a more vital menopause and a more vital old age.

According to the AEEM menopausal guidelines, a weekly combination of 2 days of strength training on alternate days with 2 days of resistance training, each lasting about 30 min, is recommended [6]. Each type of exercise stimulates the body in a different way and provides a different set of benefits. Both resistance and aerobic exercises, as well as supplementation with calcium and vitamin D, increase BMD [23,27]. When combined, these types of exercise have been found to be more effective in reducing BP and HR than aerobic exercise alone. This data was confirmed by Félix-Soriano et al. [25] who studied exercise in overweight women taking DHA-rich omega-3 supplementation. Also, Xi et al. [31] reported similar results in their study evaluating the effects of combined aerobic and resistance exercise on blood pressure (BP). Both studies suggest that these effects are more significant in overweight women, in whom body composition (fat loss and muscle mass gain) is enhanced by the combination of both types of exercise [3,25].

In addition to cardiovascular benefits [25,26], combined strength and endurance training also provides benefits in terms of increased strength and muscle mass, leading to a reduction in sarcopenia [13,27].

Ghanbari et al. [2] and Kemmler et al. [27] state that regular exercise should be started as soon as possible so that the benefits will be more numerous and significant. One of the most interesting exercise strategies is the practice of body weight exercises (tai chi, yoga, calisthenics…) because, in addition to working on strength and/or endurance, they improve flexibility and balance, thus reducing the risk of falls in aging populations [6,27].

High-Intensity Interval Training, or HIIT, can be performed in a variety of ways. HIIT running or cycling on a stationary bike is not the same as HIIT doing a CrossFit circuit [14]. CrossFit may not be the most advisable form of exercise for a menopausal woman, depending on the condition of her bones and joints. However, it is certainly an exercise that should be studied and may have benefits for menopausal women.

ElDeeb and Abdel-Aziem [30] agree that vibration platforms can produce a slight increase in BMD (especially when used while standing) and stimulate the contraction of muscle groups related to bipedal gait, which may reduce the risk of falls and fractures. However, the benefits vary depending on age: as with physical exercise, the older you get, the less benefit you get.

Increases in BMD and muscle mass with the use of vibration platforms are not greater than those obtained with exercise [30]. However, exercise offers a long list of benefits over vibration platforms. These include reduced cardiovascular risk factors, improved mood, and improved sex life. Therefore, the use of vibration platforms does not seem to be a better option than physical activity [2,27].

One of the main findings of the review was that many authors agree that physical activity has a synergistic effect with some supplements, increasing their effect [2,13,28]. For example, moderate physical activity was found to potentiate the effects of Ca on the skeleton, further reducing bone loss compared to a sedentary population taking only Ca supplements [29]. This supplement has been shown in several studies to be an effective ally alongside vitamin D in the fight against bone demineralization, even more so when consumed in the form of dairy products enriched with these and certain other minerals such as magnesium and zinc [24,32]. On the other hand, some of these supplements can be obtained by following a healthy diet in accordance with the nutritional requirements of each individual, so it would be advisable to carry out a blood test to check the levels of, for example, Ca and vitamin D before starting supplementation and, above all, try to exercise outdoors to expose the skin to sunlight and thus help to synthesize this substance [33].

On the other hand, other supplements such as WP (Whey Protein) or ZM (Zataria Multiflora), appear to be ineffective without a correct training regimen to unleash their potential, and therefore, have not been shown to provide benefits when not combined with a proper training regimen [2,28]. Other supplements with more scientific evidence, such as calcium, isoflavones, or omega-3, may increase their effect when combined with a training routine [25,34], but training is not a prerequisite for them to show their effects. In fact, omega-3 offers multiple benefits not only during the menopause years but at any stage of life, especially since a large proportion of the population has a dietary deficiency of this fatty acid [25].

One of the main limitations of the study was the lack of evidence regarding ideal routines specifically designed for this age group. Different routines were observed that should be adapted to each woman’s preferences, needs, and limitations.

HIIT training, along with vibration platforms, are understudied exercises for this stage of life. They are relatively new exercises for this stage of women’s lives, as they are mainly associated with younger people because they constitute an intense form of training. Therefore, a scarcity of literature was found to support their effectiveness.

On the other hand, it was difficult to find articles that studied supplements other than Ca and vitamin D, as these are the most widely known and consumed. There is not much evidence on the use of other supplements that promise to be tools with significant benefits and should be studied.

In addition, some of the evidence found was of lower methodological quality than expected, and half of the trials were conducted with small populations (sample size less than 100 women). In addition, this systematic review included only 10 studies, which, together with the above statement about the quality of the studies, means that our results may not be sufficient to support the conclusions.

On the other hand, in terms of physical exercise, most of the articles that studied the effect of training focused on aerobic exercise, so it would be interesting to study other types of sport practices that are understudied or generate some controversy, such as HIIT, swimming, or exercises with one’s own body weight such as calisthenics.

In response to the systematic review conducted, we believe it would be advisable to investigate other types of training such as HIIT, swimming, tai chi, yoga, and even bodyweight exercises and the use of vibration platforms, since there is some controversy and no consensus as to whether these types of exercise are recommended or not.

In the same way, supplements other than calcium, vitamin D and isoflavones should be considered, as these have already been extensively studied. There are many other options on the market that could be effective, such as omega-3, protein powders, and Zataria multiflora.

## 5. Conclusions

Based on the objectives outlined, the main conclusions of this review are as follows:

Regarding the primary objective of analysing the benefits of physical exercise and dietary supplements during menopause:

- Both strength and aerobic training, as well as supplementation with calcium and vitamin D, increase bone mineral density.
- Combined strength and resistance training provide cardiovascular benefits, increase strength and muscle mass, and reduce the risk of sarcopenia.
- Physical exercise has a synergistic effect with some supplements, enhancing their effectiveness.

Regarding the secondary objective of determining whether the relationship between dietary supplements and physical exercise mitigates the negative effects of menopause:

- Moderate physical activity enhances the effects of calcium on the skeleton, further reducing bone loss.
- Supplements such as whey protein or Zataria multiflora appear to be ineffective without an appropriate exercise regimen.
- Other supplements such as calcium, isoflavones, or omega-3 can enhance their effects when combined with a workout routine.

Regarding the secondary objective of investigating which dietary supplements and exercises may be beneficial during menopause:

- Calcium and vitamin D are effective supplements against bone demineralization, especially when consumed in enriched dairy products.
- Recommended exercises include 2 days of strength training and 2 days of aerobic resistance training per week.
- Bodyweight exercises such as yoga or tai chi are also beneficial for improving balance and flexibility.

In conclusion, the combination of supervised physical exercise along with dietary supplementation can help counteract various symptoms and negative effects of menopause in women.

## Figures and Tables

**Table 1:** Characteristics of the studies included in the results.

Authors, Year. Country	Type of Study and Duration	Sample and Characteristics	Characteristics of the Study	Objective of the Study	Main Findings	Conclusions
García-Gomáriz et al., 2018 [23]. Spain	Randomised clinical trial, 2 years.	n = 34 (IG = 17; CG = 17) Healthy menopausal women over 50 years of age supplemented with Ca (1000 mg/day) and Vit. D (880 IU)	IG; high-intensity strength training (2 sessions per week) + supplementation with Ca and Vit. D CG; brisk walking (3–5 times a week) + Ca and Vit. D supplementation	To analyse the differences in bone density level between a high-intensity strength exercise programme recommended by the AEEM and brisk walking to prevent osteoporosis in menopausal women. Both groups were supplemented with Ca and vitamin D.	The CG showed an increase in T-Score at the lumbar spine (+0.27), while the strength training group increased T-Score at both the lumbar spine (+0.47) and femoral neck (+0.28).	Although both training plans are effective in reducing the risk of osteoporosis in addition to Ca and vitamin D supplementation, strength training proved to be more effective in preventing osteoporosis at both the lumbar and femoral levels.
Giolo et al., 2018 [24]. Brazil	Randomised clinical trial, 10 weeks.	n = 32 (IG = 16; CG = 16) Healthy menopausal women aged 50–70 years undergoing a strength and endurance exercise routine 3 times a week.	IG; supplementation with 100 mg isoflavones per day + strength and endurance training. CG; placebo + strength and endurance training.	To assess the effect of isoflavone supplementation and a strength and endurance exercise routine on blood cholesterol levels, inflammatory markers, and oxidative stress.	There were no significant differences between the two groups. Oxidative stress markers and inflammatory markers were very similar at baseline and at the end of the study, except for cholesterol and interleukin-8 levels. Cholesterol levels in the placebo group decreased and interleukin-8 levels increased.	Isoflavone supplementation shows no benefit in terms of reducing cholesterol levels, inflammatory markers, or oxidative stress. However, physical exercise appears to be effective in reducing cholesterol levels; although it may increase some inflammatory markers such as interleukin-8.
Félix-Soriano et al., 2021 [25]. Spain	Randomised clinical trial, 16 weeks.	n = 124 (IG_1_ = 31; IG_2_ = 31; CG_1_ = 31; CG_2_ = 31) Overweight menopausal women aged 55–70 years supplemented with DHA-rich Omega-3 or placebo.	IG_1_; Omega 3 (1950 mg/day). IG_2_; Omega 3 (1950 mg/day) + strength training (2 times per week). CG_1_; Placebo. CG_2_; Placebo + strength training (2 times per week).	To study the benefits of DHA-rich Omega-3 supplementation and strength training in overweight menopausal women.	A reduction in body fat percentage was observed in addition to an increase in bone mineral content, muscle mass, strength, and glucose tolerance. For Omega-3 supplementation, a decrease in BP, triglyceride levels, and an increase in muscle mass were observed.	Both DHA-rich omega-3 supplementation and strength exercise show a number of cardiovascular, bone, and muscle benefits in combating menopausal complications.
Hayashi et al., 2021 [26]. Japan	Randomised clinical trial, 8 weeks.	n = 43 (IG = 27; CG = 16) Healthy menopausal women aged 45–69 years supplemented with soy isoflavones.	IG: supplementation with isoflavones (25 mg/day) + aerobic training (2–3 days per week). CG: supplementation with isoflavones (25 mg/day).	To determine whether the production of equol (a metabolite of isoflavones with oestrogen-like behaviour) by the gut microbiota exerts a synergistic effect with physical exercise in increasing arterial distensibility in menopausal women.	The increase in arterial compliance in the IG group was significantly higher (0.117 ± 0.035 mm^2^/mmHg) compared to the CG. The IG microbiota produced equol naturally with isoflavone supplementation and exercise.	Regular aerobic exercise exerts a synergistic effect with isoflavone intake, producing more equol and thus further increasing arterial compliance compared to isoflavone supplementation alone. The combination of both interventions may help to reduce cardiovascular problems such as hypertension.
Kemmler et al., 2020 [27]. Germany	Metaanalysis of studies. ≥6 months.	n = 5112 (IG = 2793; CG = 2319) Healthy menopausal women or women with osteoporosis aged 51–80 years on daily Ca and/or vitamin D supplementation.	IG; women who exercise and supplement with Ca and/or Vit. D. CG; non-exercising women who supplement with Ca and/or Vit. D.	To study the effect of different types of exercise: aerobic or anaerobic exercises with BW, DS exercises with machines, or a combination of both (BW + DS); on BMD of the femoral neck, lumbar spine, or hip in menopausal women. The training plan had to last more than 6 months and was compared with a sedentary group.	The 3 exercise programmes increased BMD in the hip, femur, and spine in different ways. Each programme increased BMD to a greater or lesser extent depending on the bone region measured. Physical exercise produces more improvements in BMD if it is practiced during early menopause, the older the age, the less benefit.	Physical exercise increases BMD significantly, but we cannot be sure that there is one particular exercise programme that increases BMD more than another.
Kuo et al., 2022 [28]. Taiwan	Metaanalysis of studies. 12–72 weeks.	n = 776 Healthy menopausal or sarcopenic women over 55 years of age on whey protein supplementation and with a sedentary lifestyle or leading a strength training routine.	IG: menopausal women supplemented with WP, 20–30 g per day + strength training for 12–72 weeks. CG: menopausal women supplemented with WP, 20–30 g per day.	To investigate the changes in strength and body composition that WP can produce in menopausal women, in combination with a strength routine or a sedentary lifestyle.	WP supplementation (20–40 g per day) had significant effects in the upper and lower body strength training groups, contributing to the increase in biceps curl strength, with a SMD: 0.6805, 95% CI, and increasing muscle mass in the lower limbs: SMD: 1.103, 95% CI.	WP is effective in combating sarcopenia and increasing strength in menopausal women if accompanied by a strength exercise programme. Taking it on its own has no significant effect on strength or improved body composition.
Nakamura et al., 2019 [29]. Japan	Randomised clinical trial, 2 years	n = 450 (IG_1_ = 150; IG_2_ = 150; CG = 150) Healthy menopausal women aged 50–75 years who engage in moderate (4 METs) or vigorous (6 METs) physical activity.	IG_1_; supplementation with 250 mg per day of Ca. IG_2_; supplementation with 500 mg per day of Ca. CG; placebo	To investigate whether a plan of vigorous (6 METs) or moderate (4 METs) physical activity can modify the effect of Ca supplementation on bone metabolism, with 250 mg, 500 mg, or placebo supplementation.	In the moderate physical activity group, namely 4 METs per week, spinal BMD decreased significantly less in the group consuming 500 mg Ca per day (−0.029 g/cm^2^, *p* = 0.042) compared to the placebo group (−0.045 g/cm^2^). In the vigorous physical activity group, there were no significant differences between supplementation and placebo groups.	Moderate physical activity modifies the effect of calcium supplementation on BMD in menopausal women, increasing its effect on bone metabolism.
Ghanbari-Niaki et al., 2020 [2]. Iran	Randomised clinical trial, 8 weeks.	n = 96 (IG = 48; CG = 48) Healthy menopausal women over 53 years of age.	IG; subdivided into 4 groups of n = 12 according to the level of training (from 0 to 85% intensity). All of them supplemented with 500 mg of ZM per day. CG; subdivided into 4 groups of n = 12 according to the level of training (from 0 to 85% intensity). None of them supplemented with ZM.	To assess the effects of strength training in menopausal women in addition to supplementation with 500 mg of ZM.	Strength training increases insulin sensitivity and plasma apelin levels (a peptide that produces cardiovascular benefits). ZM lowers blood glucose levels, so it could be synergistic with strength training.	Strength training decreases risk factors for metabolic syndrome and ZM may be helpful in lowering blood glucose levels when combined with strength training. The combination of both interventions produces cardiovascular benefits.
Hettchen et al., 2021 [13]. Germany	Randomised clinical trial, 18 months.	n = 54 (IG = 27; CG = 27) Menopausal women with osteoporosis aged 48–60 years supplemented with Ca (1000 mg/day) and Vit. D (800 IU/day).	IG; Ca and Vit. D supplementation + strength training with high-intensity weights 3 or 2 times a week. CG; Ca and Vit. D supplementation + strength training with low-intensity weights once a week.	To investigate the changes produced by strength training with weights together with Ca (1000 mg/day) and Vit. D (800 IU) supplementation in menopausal women.	The IG showed several improvements compared to the CG: a significant increase in BMD at the spine level (0.002 ± 0.018 mg/cm^2^), increased lean mass (0.39 ± 1.08 kg) and strength, decreased body fat (−1.44 ± 1.49 kg) and improved menopausal symptoms as measured by the MRS II (*p* = 0.002).	High-impact strength training is a good strategy to combat various negative effects of menopause.
ElDeeb y Abdel-Aziem. 2020 [30]. Egypt	Randomised clinical trial, 24 weeks.	n = 44 (IG = 22; CG = 22) Menopausal women aged 50–60 years with low BMD and supplemented with Ca (1200 mg/day) and Vit. D (800 IU/day).	IG; using vibration platforms 2 times a week + supplementation with Ca and Vit. D CG; no use of vibration platforms + supplementation with Ca and Vit. D	To study the effect of vibration platform training on muscle work and BMD in menopausal women consuming 1200 mg Ca per day and 800 IU Vit D per day.	The IG showed a greater increase in BMD at the lumbar and femoral levels than the CG (*p* > 0.05). It also showed increased strength in the muscle groups involved in gait, which was not reflected in the CG.	Vibration platform therapy increases hip, knee, and ankle strength and increases BMD in the lumbar spine and femur in menopausal women.

IG: Intervention Group, CG: Control Group, AEEM: Spanish Association for the Study of the Menopause, BW: Body Weight, DS: Dynamic Strength, BMD: Bone Densitometry, WP: whey protein, SMD: Standardised Measurement Difference, MET: Metabolic Index Measurement Unit, ZM: Zataria Multiflora, MRS: Menopause Rating Scale. Source: own elaboration.

**Table 2:** Evaluation of the methodological quality of the tests included in the work by means of the scale PEDro.

Authors	Criteria										Total	Methodological Quality Value
	2	3	4	5	6	7	8	9	10	11		
Nakamura et al. [29]	+	-	+	-	-	-	+	+	+	+	6	Good
García-Gomáriz et al. [23]	+	+	-	+	-	-	-	+	-	+	5	Regular
Félix-Soriano et al. [25]	+	-	+	-	-	-	-	-	+	+	4	Regular
Hettchen et al. [13]	+	+	+	-	-	+	+	+	+	+	9	Excellent
Ghanbari-Niaki et al. [2]	+	-	+	-	-	-	+	+	+	+	7	Good
ElDeeb y Abdel-Aziem. [30]	+	+	+	-	-	-	+	-	+	+	6	Good
Giolo et al. [24]	+	+	+	-	-	-	+	+	+	+	7	Good
Hayashi et al. [26]	+	-	+	-	-	-	+	+	+	+	6	Good

+: Yes; -: No. Source: own elaboration.

**Table 3:** Methodological quality assessment of the included meta-analyses using the AMSTAR-2 scale.

Authors	1	2	3	4	5	6	7	8	9	10	11	12	13	14	15	16	General Confidence Value
Kemmler et al. [27]	+	+	-	+ partial	+	+	+	+ partial	+	+	+	-	+	+	+	+	Medium
Kuo et al. [28]	-	+	-	+ partial	+	+	+	+ partial	+ partial	-	+ partial	-	+	+	+	+	Medium

+: Yes; -: No. Source: own elaboration.

## Data Availability

Data are available on request from the corresponding author.

## Supplementary Materials

The following supporting information can be downloaded at: https://www.mdpi.com/article/10.3390/jcm12237271/s1, Table S1: Decs/mesh terms used in the search; Table S2: Search strategy and information sources.
