# Supplementary material for: Physical Exercise and Dietary Supplementation in Middle-Aged and Older Women: A Systematic Review
Source: J Clin Med. 2023 Nov 23;12(23):7271. doi: 10.3390/jcm12237271 (PMC10707386; doi:10.3390/jcm12237271)
Supplement: Supplementary file 1 [file jcm-12-07271-s001.zip › Table S1.pdf]

TERMS USED IN THE SEARCH.

Table S1. Decs/mesh terms used in the search.

Descriptors in Health Sciences (DeCS) - Spanish	Medical Subject Headings (MeSH)	Controlled language	Natural language
Menopausia	Menopause	Menopause	Change of life female
Ejercicio	Exercise	Exercise	Exercises OR "Physical Activity" OR "Activities Physical" OR "Activity Physical" OR "Physical Activities" OR "Exercise Physical" OR "Exercises Physical" OR "Physical Exercise" OR "Physical Exercises" OR "Acute Exercise" OR "Acute Exercises" OR "Exercise Acute" OR "Exercises Acute" OR "Exercise Isometric" OR "Exercises Isometric" OR "Isometric Exercises" OR "Isometric Exercise" OR "Exercise Aerobic" OR "Aerobic Exercise" OR "Aerobic Exercises" OR "Exercises Aerobic" OR "Exercise Training" OR "Exercise Trainings" OR "Training Exercise"
Suplementos dietéticos	Dietary supplements	Dietary supplements	"Dietary Supplement" OR "Supplements Dietary" OR "Dietary Supplementations" OR "Supplementations Dietary" OR "Food Supplementations" OR "Food Supplements" OR "Food Supplement" OR "Supplement Food" OR "Supplements Food" OR "Nutraceuticals" OR "Nutraceutical" OR "Nutriceuticals" OR "Nutriceutical" OR "Neutraceuticals" OR "Neutraceutical" OR "Herbal Supplements" OR "Herbal Supplement" OR "Supplement Herbal" OR "Supplements Herbal"

Source: self made.
