# Supplementary material for: Physical Exercise and Dietary Supplementation in Middle-Aged and Older Women: A Systematic Review
Source: J Clin Med. 2023 Nov 23;12(23):7271. doi: 10.3390/jcm12237271 (PMC10707386; doi:10.3390/jcm12237271)
Supplement: Supplementary file 1 [file jcm-12-07271-s001.zip › Table S2.pdf]

Table S2. Search strategy and information sources.

DATABASES AND FILTERS APPLIED	SEARCH STRATEGY	DATE	OUTCOMES (Found), selected
PUBMED<br><br>FILTERS: Clinical Trial, Meta-Analysis, Randomized Controlled Trial, in the last 5 years.	Menopause OR (natural language) AND “Dietary Supplements” OR (natural language) AND Exercise OR (natural language):<br><br>((("menopause"[MeSH Terms] AND "exercise"[MeSH Terms]) OR ("Exercises"[Title/Abstract] OR "Physical Activity"[Title/Abstract] OR "Activities Physical"[Title/Abstract] OR "Activity Physical"[Title/Abstract] OR "Physical Activities"[Title/Abstract] OR "Exercise Physical"[Title/Abstract] OR "Exercises Physical"[Title/Abstract] OR "Physical Exercise"[Title/Abstract] OR "Physical Exercises"[Title/Abstract] OR "Acute Exercise"[Title/Abstract] OR "Acute Exercises"[Title/Abstract] OR "Exercise Acute"[Title/Abstract] OR "Exercises Acute"[Title/Abstract] OR "Exercise Isometric"[Title/Abstract] OR "Exercises Isometric"[Title/Abstract] OR "Isometric Exercises"[Title/Abstract] OR "Isometric Exercise"[Title/Abstract] OR "Exercise Aerobic"[Title/Abstract] OR "Aerobic Exercise"[Title/Abstract] OR "Aerobic Exercises"[Title/Abstract] OR "Exercises Aerobic"[Title/Abstract] OR "Exercise Training"[Title/Abstract] OR "Exercise Trainings"[Title/Abstract] OR "Training Exercise"[Title/Abstract])) AND "dietary supplements"[MeSH Terms] AND ("Dietary Supplement"[Title/Abstract] OR "Supplements Dietary"[Title/Abstract] OR "Dietary Supplementations"[Title/Abstract] OR "Supplementations Dietary"[Title/Abstract] OR "Food Supplementations"[Title/Abstract] OR "Food Supplements"[Title/Abstract] OR "Food Supplement"[Title/Abstract] OR "Supplement Food"[Title/Abstract] OR "Supplements Food"[Title/Abstract] OR "Nutraceuticals"[Title/Abstract] OR "Nutraceutical"[Title/Abstract] OR "Nutriceuticals"[Title/Abstract] OR "Nutriceutical"[Title/Abstract] OR "Neutraceuticals"[Title/Abstract] OR "Neutraceutical"[Title/Abstract] OR "Herbal	11/27/2022	(16), 5

	Supplements"[Title/Abstract] OR "Herbal Supplement"[Title/Abstract] OR "Supplement Herbal"[Title/Abstract] OR "Supplements Herbal"[Title/Abstract])) AND ((y_5[Filter]) AND (clinicaltrial[Filter] OR meta-analysis[Filter] OR randomizedcontrolledtrial[Filter]		
WOS<br><br>FILTERS: Clinical Trial, Article, publication years 2018-2022, NOT review article OR meeting OR abstract OR other.	Menopause OR (natural language) AND “Dietary Supplements” OR (natural language) AND Exercise OR (natural language):<br><br>((TS=(menopause OR “Change of Life Female”)) AND TS=(exercise OR Exercises OR “Physical Activity” “Activities Physical” OR “Activity Physical” OR “Physical Activities” OR “Exercise Physical” OR “Exercises Physical” OR “Physical Exercise” OR “Physical Exercises” OR “Acute Exercise” OR “Acute Exercises” OR “Exercise Acute” OR “Exercises Acute” OR “Exercise Isometric” OR “Exercises Isometric” OR “Isometric Exercises” OR “Isometric Exercise” OR “Exercise Aerobic” OR “Aerobic Exercise” OR “Aerobic Exercises” OR “Exercises Aerobic” OR “Exercise Training” OR “Exercise Trainings” OR “Training Exercise”)) AND TS=("Dietary Supplements" OR “Dietary Supplement” OR “Supplements Dietary” OR “Dietary Supplementations” OR “Supplementations Dietary” OR “Food Supplementations” OR “Food Supplements” OR “Food Supplement” OR “Supplement Food” OR “Supplements Food” OR “Nutraceuticals” OR “Nutraceutical” OR “Nutriceuticals” OR “Nutriceutical” OR “Neutraceuticals” OR “Neutraceutical” OR “Herbal Supplements” OR “Herbal Supplement” OR “Supplement Herbal” OR “Supplements Herbal”) and 2022 or 2021 or 2020 or 2019 or 2018 (Publi cation Years) AND Article or Clinical Trial (Document Types) NOT review article OR meeting (Document Types)	11/29/2022	(47), 3
SCOPUS<br><br>FILTERS: Article, years 2018 – 2022.	Menopause OR (natural language) AND “Dietary Supplements” OR (natural language) AND Exercise OR (natural language):<br><br>(TITLE-ABS-KEY ("dietary supplements" OR "dietary supplement" OR "supplements dietary" OR "dietary supplementations" OR "supplementations dietary" OR "food supplementations" OR "food supplements" OR "food supplement" OR "supplement food" OR "supplements food" OR "nutraceuticals" OR "nutraceutical" OR "nutric	12/14/2022	(27), 2

	euticals" OR "nutriceutical" OR "neutraceuticals" OR "neutraceutical" OR "herbal supplements" OR "herbal supplement" OR "supplement herbal" OR "supplements herbal")) AND (TITLE-ABS-KEY (menopause OR "change of life female")) AND (TITLE-ABS-KEY (exercise OR exercises OR "physical activity" "activities physical" OR "activity physical" OR "physical activities" OR "exercise physical" OR "exercises physical" OR "physical exercise" OR "physical exercises" OR "acute exercise" OR "acute exercises" OR "exercise acute" OR "exercises acute" OR "exercise isometric" OR "exercises isometric" OR "isometric exercises" OR "isometric exercise" OR "exercise aerobic" OR "aerobic exercise" OR "aerobic exercises" OR "exercises aerobic" OR "exercise training" OR "exercise trainings" OR "training exercise")) AND (LIMIT-TO (PUBYEAR , 2022) OR LIMIT-TO (PUBYEAR , 2021) OR LIMIT-TO (PUBYEAR , 2020) OR LIMIT-TO (PUBYEAR , 2019) OR LIMIT-TO (PUBYEAR , 2018)) AND (LIMIT-TO (DOCTYPE , "ar"))		
COCHRANE<br><br>FILTERS: Custom Range 2018 to 2022.	Menopause OR (natural language) AND “Dietary Supplements” OR (natural language) AND Exercise OR (natural language):<br><br>MeSH descriptor: (Menopause) OR (Change of Life Female): ti,ab,kw AND MeSH descriptor (Exercise) OR (Exercises OR Physical Activity OR Activities Physical OR Activity Physical OR Physical Activities OR Exercise Physical OR Exercises Physical OR Physical Exercise OR Physical Exercises OR Acute Exercise OR Acute Exercises OR Exercise Acute OR Exercises Acute OR Exercise Isometric OR Exercises Isometric OR Isometric Exercises OR Isometric Exercise OR Exercise Aerobic OR Aerobic Exercise OR Aerobic Exercises OR Exercises Aerobic OR Exercise Training OR Exercise Trainings OR Training Exercise):ti,ab,kw AND MeSH descriptor: (Dietary Supplements) OR (Dietary Supplement OR Supplements Dietary OR Dietary Supplementations OR Supplementations Dietary OR Food Supplementations OR Food Supplements OR Food Supplement OR Supplement Food OR Supplements Food OR Nutraceuticals OR Nutraceutical OR Nutriceuticals OR Nutriceutical OR Neutraceuticals OR Neutraceutical OR Herbal Supplements OR Herbal Supplement OR Supplement Herbal OR Supplements Herbal):ti,ab,kw	12/15/2022	(14), 0

TOTAL			(104), 10
